# Nasopharyngeal microbiota is influenced by agricultural air pollution in individuals with and without COPD

**DOI:** 10.1038/s41598-025-00242-9

**Published:** 2025-05-05

**Authors:** Mari-Lee Odendaal, Julia Taenzer, Myrna M. T. de Rooij, Sjoerd Kuiling, Debby Bogaert, Eelco Franz, Lidwien A. M. Smit

**Affiliations:** 1 Centre for Infectious Disease Control, National Institute for Public Health and the Environment (RIVM), Bilthoven, The Netherlands; 2 Institute for Risk Assessment Sciences (IRAS), Utrecht University, Utrecht, The Netherlands; 3 Department of Paediatric Immunology and Infectious Diseases, Wilhelmina Children’s Hospital/University Medical Center Utrecht, Utrecht, The Netherlands; 4 Centre for Inflammation Research, Queen’s Medical Research Institute, University of Edinburgh, Edinburgh, UK

**Keywords:** Livestock-related air pollution, COPD, Nasopharyngeal microbiota, Ammonia exposure, Anaerobic bacteria, 16S rRNA amplicon sequencing, Molecular biology, Environmental sciences, Chronic obstructive pulmonary disease

## Abstract

Respiratory health in chronic obstructive pulmonary disease (COPD) is influenced by environmental factors such as air pollution, yet the role of the airway microbiota in this relationship remains unclear. We investigated the association between exposure to air pollution from livestock farms and the nasopharyngeal microbiota in individuals with COPD compared to healthy control subjects. The study included nasopharyngeal swabs from 186 currently non-smoking participants in the Netherlands, including 65 individuals with COPD and 121 without from a regional rural cohort. Additionally, 116 individuals from a population-wide cohort were included as national controls. Samples were taken at three time points over 12 weeks. The nasopharyngeal microbiota was studied using 16 S rRNA gene-based sequencing for all baseline samples and a random selection of 6-weeks and 12-weeks samples. Dispersion models were used to determine the average concentrations of livestock-related PM_10_, endotoxin, and ammonia at the participants’ home addresses. Individuals with COPD had a higher absolute abundance of anaerobic bacteria, such as *Peptoniphilus*, *Anaerococcus*, *Finegoldia magna*, and *Prevotella*. Importantly, residential exposure to ammonia was identified as the most important driver of the microbial community composition, explaining 6.6% of the variation in nasopharyngeal microbiota in individuals with COPD. Higher ammonia concentrations were associated with decreased levels of key commensals and increased abundance of anaerobic bacteria. Furthermore, individuals living in areas with high livestock density exhibited greater microbial diversity compared to the broader national population. The study highlights the influence of residential exposure to livestock-related air pollution, particularly ammonia, on nasopharyngeal microbiota composition in individuals with COPD. Our findings suggest that environmental factors significantly impact microbial communities and underscore the potential role of anaerobic bacteria in COPD pathology. Future research should further investigate the mechanisms by which environmental air pollutants affect microbial communities and explore potential interventions to mitigate their effects on respiratory health.

**Supplementary Information:**

The online version contains supplementary material available at 10.1038/s41598-025-00242-9.

## Introduction

Chronic obstructive pulmonary disease (COPD) represents a significant public health challenge, exerting a substantial burden on individuals, healthcare systems, and society at large. Characterized by persistent respiratory symptoms and airflow limitation, COPD is a leading cause of morbidity and mortality worldwide, contributing to considerable healthcare costs and diminished quality of life for those affected^1–3^. It is well-established that smoking is the leading cause of COPD. However, other critical risk factors contributing to the disease pathogenesis are less well understood^4^.

To advance our understanding of the factors contributing to COPD, recent research has increasingly focused on the complex interplay between environmental exposures and respiratory health. Growing evidence suggests that both occupational^5,6^ and residential^7–9^ exposures related to livestock farming play a role in the development and progression of COPD. Among others, COPD patients living near livestock farms experienced a higher rate of exacerbations^7^ and more severe respiratory symptoms^8^ compared to those living further away. Individuals living near livestock farms are more exposed to farm emissions consisting of complex mixtures of gases like ammonia (NH_3_) and particles predominantly from biological origin. Research has shown clear elevations in residential exposures to particulate matter (PM), ammonia (NH_3_) and endotoxins (a potent bacterial toxin), which have been linked to adverse health effects^9–14^. Several studies have linked increased NH_3_ levels^9,11,12^ and short-term exposure to livestock farming endotoxins^13^ to reduced lung function in non-farming residents. While respiratory infections and environmental exposures are known to influence COPD pathogenesis^15,16^, the mechanisms underlying these adverse health effects remain poorly understood. Interestingly, living near livestock farms, as sources of chemical and microbial air pollution, could impact respiratory tract microbiota and contribute to respiratory health issues.

The respiratory microbiome plays a crucial role in maintaining respiratory health and modulating disease processes, including COPD^17,18^. By interacting with the mucosal immune system, it provides a barrier against harmful microorganisms^19–21^. Specifically, the upper respiratory tract (URT) microbiome acts as a gatekeeper, preventing harmful pathogens from entering the lower airways and maintaining respiratory health^19,22–24^. Due to its direct exposure to the external environment, the URT must continuously adapt to changes in both host and environmental factors, which in turn impact health outcomes^25^. Interestingly, previous research has successfully connected the URT microbiota with respiratory health outcomes and exposure to livestock farms, though evidence remains limited^26,27^. Consequently, investigating the URT microbiota can provide valuable insights into the link between livestock-related air pollution and COPD.

The Netherlands serves as an ideal setting for studying the relationship between air pollution from livestock farming and COPD due to the close coexistence of intensive farming and residential areas. This raises notable health concerns, particularly for individuals with COPD who are more vulnerable to environmental pollutants because of their compromised airway performance. Building on our previous research on the oropharyngeal microbiota^27^, this study aims to investigate the nasopharyngeal microbiota in individuals with COPD and its association with residential proximity to livestock farming. Specifically, we aimed to: (i) characterize the stability of the nasopharyngeal microbiota over time; (ii) analyse the differences in the nasopharyngeal microbiota between individuals with COPD and healthy controls in relation to residential exposure; and (iii) compare the nasopharyngeal microbial communities of individuals living in livestock-dense areas to those in the broader population. Ultimately, a deeper understanding of the potential impact of livestock farming on nasopharyngeal microbiota and its implications for COPD is crucial for identifying the environmental factors that contribute to respiratory diseases and thereby guiding effective public health policies.

## Methods

### Study and population design

Participants of this study were selected from the cross-sectional Dutch Livestock Farming and Neighbouring Residents’ Health study (VGO) population, 2369 individuals in total. Information on the design, enrolment and medical procedures used for the VGO population have been described previously^12^. Participants with COPD were selected by a lung function specialist based on their spirometry value and curves or reported a COPD diagnosis by a physician^27^. The control group was randomly selected from individuals without asthma, without a history of smoking, and without COPD, all with healthy lung function. Additionally, a random selection of nasopharyngeal samples was obtained from the population-wide cohort Pienter3^25^. These samples were drawn from individuals aged 18–70, with current smokers excluded from the selection. The Pienter3 participants served as national controls, representing a mix of urban and rural areas across the Netherlands (collected in 2015–2016). In contrast, the VGO participants were specifically drawn from a high livestock-density rural area in North Brabant and Limburg. This distinction enables a clearer comparison between rural livestock-dense areas and the broader national population. Informed written consent was obtained from all participants of the study.

### Sampling procedure

Participant information and nasopharyngeal swabs were collected during home visits in 2015–2016. Samples were included from a total of 221 individuals, including 81 individuals with COPD (VGO cases) and 140 individuals without (VGO controls; Table S1). Samples from the VGO population were collected from all participants at three distinct time points: initially at baseline (T0), followed by additional collections after 6 weeks (T1) and 12 weeks (T2). Copan eSwabs were used for sample collection and stored in 1 ml liquid Amies Medium (483CE, Copan Diagnostics Inc., CA) for transport^28^. All T0 samples, together with a random selection of 12 T1 and 12 T2 samples, were processed through DNA extraction and sequencing. In addition, field blanks (air swabs) were taken each day of sampling and processed along with laboratory controls.

### DNA isolation and 16S-rRNA gene sequencing

Laboratory procedures for DNA isolation and gene amplification were performed as described in our protocol^28,29^. DNA was extracted from nasopharyngeal swabs using Mag Mini DNA Isolation Kit with slight modifications to enhance reliability in extracting low biomass DNA^30^. Each isolation run included a 200 µl aliquot of a 10^3^ dilution of ZymoBIOMICS microbial community standard as a positive control, along with two negative controls consisting of lysis buffer only (referred to as DNA isolation blanks). The samples were mechanically disrupted for 2 minutes at 3500 oscillations/minute using a Mini-Beadbeater-24 (Biospec Products). Between each bead-beating, samples were transferred onto ice for 2 minutes. In line with standard protocol, using the 515F (5’-GTGCCAGCMGCCGCGGTAA-3’)/806R (5’-GGACTACHVGGGTWTCTAAT-3’)-primer pair, amplification of the hypervariable region 4 (V4) of the 16 S-rRNA-gene was conducted^31^. Following this, the amplicon pools were purified by two consecutive purifications using 0.9x AMPure XP magnetic beads. Following purification, the MiSeq reagent kit V3 (600-cycle) was used to sequence the pools on an Illumina MiSeq instrument (Illumina Inc., San Diego, CA, US). To ensure a valid sequencing process and identify possible contaminants, both the positive (ZymoBIOMICS microbial community DNA standard) and negative (sequencing) controls were incorporated. The mock communities were inspected for irregular microbial profiled across the DNA isolation and MiSeq-runs.

### Bioinformatics

An in-house bioinformatics pipeline was utilized for the processing of paired-end reads using DADA2^32^ (v1.16.0; maxEE = 2; truncLen = 200/150). Chimeras were detected and eliminated using the ‘consensus’ method. Assignment of taxonomy was conducted using the naïve Bayesian classifier and the Silva v138 (Version 2; August 2020) reference database^33^.

### Residential exposure to livestock farming

For all individuals, exposure proxies, like the number of farms in the vicinity, were computed using Geographic Information System (GIS) software (ArcGIS; version 10.2.2, Esri) using geolocated livestock farm data combined with geocoded residential addresses, as previously described^12^. In addition, more refined exposure parameters were available for VGO participants as obtained from earlier research. Dispersion modelling was applied to estimate annual average residential exposure to livestock farming emitted PM_10_ and endotoxin^10^. In brief, this dispersion model is based on the Gaussian plume model and implements the Netherlands New National Model. This model predicts dispersion of livestock farming emitted PM_10_ and endotoxin using data on source-level (e.g. farm-type, number of animals) and data relevant for dispersion in the surroundings of the source (e.g. local terrain roughness and meteorological conditions). Regional week-average concentrations of NH_3_ in the week prior to sampling were included as previously described^12^. Measured ambient NH_3_ concentrations were obtained from two rural background stations from the Dutch Air Quality Monitoring Network situated within the study area. For statistical analyses, air pollution data was split into tertiles designating low, medium and high exposure levels to the respective air pollutant.

### Pre-processing

The raw dataset included 15,914,054 sequences and 14,656 amplicon sequence variants (ASVs) from a total of 385 samples (VGO and Pienter3). Prior to the data analysis, several filtering steps were conducted to remove contaminated and low-quality samples (Fig. S1A). Off-target reads and taxa belonging to the phylogenetic groups ‘Mitochondria’, ‘Chloroplast’, ‘Archaea’ and ‘Eukaryota’ were removed^34^. We renamed the ASVs using the lowest identified taxonomic rank, followed by the rank number (based on relative abundance) in brackets.

We identified potential contaminants separately for the two populations (VGO and Pienter3) using for each cohort the ‘combined’ method of the “decontam” R-package (version 1.10.0) (isContaminant()-function)^35^ including their respective DNA isolation blanks. Using this approach, we identified 67 and 45 contaminant ASVs in the VGO and Pienter3 population, respectively, which we combined in a single list (*n* = 107). These potential contaminant ASVs were also identified as the most abundant ASVs in the blanks and included *Comamonadaceae* (6), *Ralstonia* (14), *Thermus* (7) and *Caulobacter* (8) (Fig. S1B), which are all common reagent and laboratory contaminants^36^.

Additionally, we manually identified and removed low abundant ASVs that had an irregular relative abundance across isolation runs. Finally, a total of 41 samples (11.63%) with a bacterial density lower than 0.095 pg/ul as determined by qPCR or less than 10,000 reads were removed (Fig. S1C,D), retaining a total of 344 nasopharyngeal samples following all pre-processing steps. For downstream analyses, we included ASVs with a relative abundance of at least 0.1% in a minimum of 2 samples, except when calculating alpha diversity indices.

### Data analysis and visualization

All analyses were conducted in R v4.3.2 using RStudio Server v2023.3.0.386. Statistical tests were two-sided, and *p*-values were adjusted for multiple comparisons using the Benjamini-Hochberg (BH) method (*q*-values).

The main analyses were performed using only the baseline (T0) nasopharyngeal samples. Stability analyses, were conducted using samples from individuals for whom follow-up samples (T1 or T2) were sequenced. The stability of the nasopharyngeal microbiota over time was determined using Wilcoxon signed-rank tests, with Bray-Curtis dissimilarities/distances serving as a measure of community composition similarity. We determined whether community composition of the nasopharyngeal samples differed more ‘between’ individuals than ‘within’ individual. “Within” referring to the community composition from a singular individual at different time points and “between” referring to samples from different individuals at different time points.

We used the Pearson’s Chi-square test to identify potential collinearity among all variables (Table S2). Additionally, we conducted univariable PERMANOVA (Fig. S2; outcome: community composition) and linear regression (Table S3; outcomes: bacterial density/microbial diversity) to identify potential confounders for adjustment in our multivariable models, especially focusing on variables with *p*-values < 0.2. This threshold was only applied to identify potential confounder. Consequently, we adjusted for sampling season, age category, gender, atopy, antibiotic use, education level, and smoking status.

Bray-Curtis dissimilarity matrices were generated by implementing the “*vegan*” R-package to illustrate the beta diversity patterns of the microbial communities^37^, with the Hellinger transformation applied. Additionally, Aitchison distances were calculated to account for the compositional nature of the data and to support the Bray-Curtis-based findings. Permutational multivariate analysis of variance (PERMANOVA) was implemented to determine the explained variance (R^2^) of population and livestock-related exposure. PERMANOVAs were performed only when there were at least 10 participants in each category being investigated.

To analyse alpha diversity, we rarefied the count data to account for differences in sequencing depth, using the lowest number of reads (min reads = 14,507). The “*microbiome*” R-package was employed to calculate the Shannon index and observed ASVs. Additionally, we applied a log_2_ transformation to bacterial density to visualize the overall bacterial load in the samples. Linear models were used to assess differences between VGO subjects with COPD vs VGO subjects without COPD, VGO subjects without COPD vs. Pienter3 subjects, and the effect of the various livestock farm related exposure variables in the overall population and stratified by population.

Two differential abundance analysis methods, MaAsLin2 and ANCOM-BC2, were implemented to investigate the association between COPD and livestock farm exposure (number of farms, endotoxin, PM_10_, NH_3_) with the absolute abundance of the most prevalent ASVs. The absolute abundance of the ASVs was calculated by multiplying the quantitative polymerase chain reaction (qPCR) measurements with the relative abundance. We focused on the top 29 nasopharyngeal ASVs, each with over 1% relative abundance in at least 5% of the samples, collectively accounting for 82% of the total relative abundance in the nasopharynx. This approach was chosen to focus on ASVs that were sufficiently represented across samples, ensuring meaningful and robust results. We applied the default significance threshold of a *q*-value < 0.25.

Due to the weak association of endotoxin (*p* = 0.032) and NH_3_ (*p* < 0.001) with the season of sampling, we performed a sensitivity analysis to evaluate the potential impact of seasonal variation on our findings. This involved running multiple multivariable models, testing NH_3_, endotoxin, and season independently, as well as models combining endotoxin with season and NH_3_ with season. We then compared the estimates for endotoxin, NH_3_, and season across these different models.

## Results

### Study population characteristics

Out of 361 individuals included in the sample selection, samples from 320 (89%) successfully passed quality control and all pre-processing steps (Table S1). Furthermore, active smokers and Pienter3 controls with a history of lung disease were excluded, resulting in a total of 302 individuals used in the analyses. Our analyses included 65 VGO (COPD) cases, 121 VGO (rural) controls, and 116 Pienter3 (national) controls. The study population consisted of 44% women, and 76% of participants were older than 51.3 years. Participants with COPD were notably older than control subjects (Table S2; *p*-value = 0.004). Sample collection predominantly occurred during spring (Table 1; 30%) and summer (30%). Most individuals had a medium education level (43%) and, since current smokers were excluded, were either ex-smokers (54%) or never smokers (46%). Among the individuals with COPD, the majority had mild to moderate severity according to the COPD GOLD stage, with only 1 classified as severe. Lung medication use was, as expected, common among COPD cases, with 35% using such medications during their intake medical exam. Atopy was present in 30% of the population. Respiratory symptoms were reported by 17% of VGO cases, 24% of VGO controls, and 33% of Pienter3 controls. The lower prevalence of symptoms observed in COPD cases within this study may be attributed to the predominance of mild COPD cases, potentially further managed through medication control. Antibiotic use was primarily noted in individuals with COPD, with 14% reporting antibiotic use within four weeks of sample collection.

Among the VGO population, 35% were raised on a farm. The proximity of residences to livestock farms differed between the three populations. Specifically, only 29% of VGO controls did not live within 500 m of a farm, while 71% of Pienter3 controls lived beyond this distance. Regarding poultry farms, 51% of VGO individuals with COPD and 60% of the controls had poultry farms within 1 km of their residence, compared to only 8.6% of Pienter3 controls. This is expected, given the distinct recruitment regions: VGO participants were recruited from an area with high-density livestock farming, while the Pienter3 cohort represents a national sample from various regions across the Netherlands. Additionally, individuals with COPD had lower exposure to NH_3_, with 27% of individuals falling into the high category compared to 38% for VGO controls (Table 1; *p*-value = 0.041).

**Table 1:** Study population characteristics of VGO (COPD) cases, VGO (rural) controls and Pienter3 (national) controls sampled for nasopharyngeal microbiota analysis and retained after pre-processing.

Characteristics	VGO cases (*N* = 65)	VGO controls (*N* = 121)	Pienter3 controls, (*N* = 116)	Sum	*p*-value
Gender - Female	26 (37%)	59 (49%)	51 (44%)	134 (44%)	0.299
Age category					0.004
< 51.3	7 (11%)	33 (27%)	32 (28%)	72 (24%)	
51.3–60.8	15 (23%)	31 (26%)	36 (31%)	82 (27%)	
60.8–66.1	19 (29%)	34 (28%)	16 (14%)	69 (23%)	
> 66.1	24 (37%)	23 (19%)	32 (28%)	79 (26%)	
Season					< 0.001
Summer	15 (23%)	46 (38%)	31 (27%)	92 (30%)	
Fall	15 (23%)	6 (5.0%)	27 (23%)	48 (16%)	
Winter	19 (29%)	32 (26%)	19 (16%)	70 (23%)	
Spring	16 (25%)	37 (31%)	39 (34%)	92 (30%)	
Education level					0.019
Low	15 (23%)	28 (23%)	44 (40%)	87 (29%)	
Medium	35 (54%)	56 (46%)	37 (33%)	128 (43%)	
High	15 (23%)	37 (31%)	30 (27%)	82 (28%)	
Smoke status					0.211
Never smoker	25 (38%)	54 (45%)	60 (52%)	139 (46%)	
Ex-smoker	40 (62%)	67 (55%)	56 (48%)	163 (54%)	
COPD gold					< 0.001
No COPD	14 (25%)	113 (100%)	X	127 (75%)	
Mild	26 (46%)	X	X	26 (15%)	
Moderate	15 (27%)	X	X	15 (8.9%)	
Severe	1 (1.8%)	X	X	1 (0.6%)	
Used lung medication	23 (35%)	1 (0.8%)	X	24 (13%)	< 0.001
Atopy	16 (25%)	32 (27%)	42 (36%)	90 (30%)	0.172
Respiratory symptoms	11 (17%)	29 (24%)	38 (33%)	78 (26%)	0.044
Used antibiotics	9 (14%)	4 (3.3%)	8 (9.4%)	21 (7.7%)	0.029
Farm child	25 (38%)	39 (33%)	X	64 (35%)	0.514
Farms within 500 m					< 0.001
No farms	22 (34%)	35 (29%)	82 (71%)	139 (46%)	
1–2	22 (34%)	54 (45%)	29 (25%)	105 (35%)	
> 2	21 (32%)	32 (26%)	5 (4.3%)	58 (19%)	
Farms within 1 km					< 0.001
0–16	55 (85%)	105 (87%)	116 (100%)	276 (91%)	
> 16	10 (15%)	16 (13%)	0 (0%)	26 (8.6%)	
Poultry farm within 1 km	33 (51%)	72 (60%)	10 (8.6%)	115 (38%)	< 0.001
Endotoxin					0.137
Low	27 (42%)	40 (33%)	X	67 (36%)	
Middle	22 (34%)	33 (28%)	X	55 (30%)	
High	16 (25%)	47 (39%)	X	63 (34%)	
PM_10_					0.202
Low	26 (40%)	41 (34%)	X	67 (36%)	
Middle	23 (35%)	34 (28%)	X	57 (31%)	
High	16 (25%)	45 (38%)	X	61 (33%)	
NH_3_					0.041
Low	30 (47%)	34 (28%)	X	64 (35%)	
Middle	17 (27%)	41 (34%)	X	58 (32%)	
High	17 (27%)	45 (38%)	X	62 (34%)	

### Nasopharyngeal microbial community stability over time

**Fig. 1:**
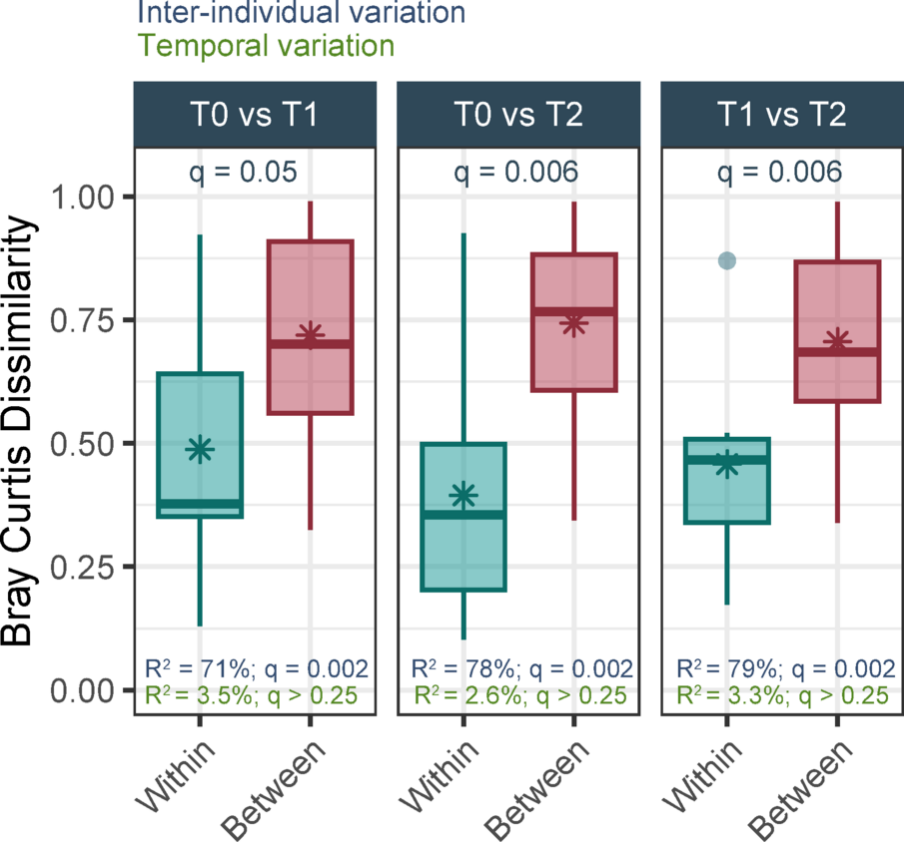
Stability of the nasopharyngeal microbiota over time. Bray-Curtis dissimilarities between samples collected at baseline (T0), 6 (T1) and 12 weeks (T2) “within” and “between” individuals. Boxplots represent the 25th and 75th percentiles (lower and upper boundaries of boxes, respectively), the median (middle horizontal line), and measurements that fall within 1.5 times the interquartile range (IQR; distance between the 25th and 75th percentiles; whiskers). Mean Bray-Curtis dissimilarities are indicated by the asterisks. *P*-values determined by implementing the Wilcoxon signed-rank test. PERMANOVA analysis was used to determine the inter-individual and temporal variation.

The dataset included 1043 ASVs with a relative abundance of ≥ 0.1% in at least 2 samples, covering 16 phyla, 27 classes, 69 orders, 126 families, 267 genera and 336 species. The nasopharyngeal microbiota of individuals with COPD and those without was similar to that of a typical nasopharyngeal community composition previously reported, including high abundances of *Moxarella*, *Staphylococcus*, *Corynebacterium* and *Dolosigranulum*^25^. The composition of the nasopharyngeal microbiota over time “within” individual had a lower heterogeneity (Fig. 1; Fig. S3; mean Bray-Curtis dissimilarity ${\bar{\text{x}}}$ = 0.44) than “between” different individuals (${\bar{\text{x}}}$ = 0.72). To better understand the impact of inter-individual and temporal variation on microbial community composition, we performed PERMANOVA analyses across all timepoint comparisons. Inter-individual differences explained a large proportion of the variance, ranging from 71 to 79% (*q*-values = 0.002). The highest explained variance was observed between T0 and T2, consistent with this being the longest time interval (Fig. 1). In contrast, time point did not significantly contribute to variation in community composition. From this, we conclude that the microbial community composition is primarily shaped by inter-individual differences rather than temporal changes.

### Alpha and beta diversity of nasopharyngeal microbiota in individuals exposed to livestock farms with and without COPD.

We assessed the explained variance in community composition attributable to the various host and environmental characteristics using univariable PERMANOVA’s. Within the overall VGO population, the three most important drivers were season (*R*^2^ = 2.8%; *p*-value = 0.005), age (*R*^2^ = 2.5%; *p*-value = 0.022) and NH_3_ residential exposure (Fig. S2; *R*^2^ = 2.4%; *p*-value = 0.004), respectively. This pattern persisted when stratifying by COPD status. Among the controls, the leading factors were season (*R*^2^ = 3.4%; *p*-value = 0.058), age (*R*^2^ = 3.0%; *p*-value = 0.123) and NH_3_ exposure (*R*^2^ = 2.3%; *p*-value = 0.079). Interestingly, in individuals with COPD, the primary drivers were NH_3_ exposure (*R*^2^ = 6.6%; *p*-value = 0.002), season (*R*^2^ = 5.4%; *p*-value = 0.187) and farms within 500 m of the participant’s home address (*R*^2^ = 5.2%; *p*-value = 0.013).

**Fig. 2:**
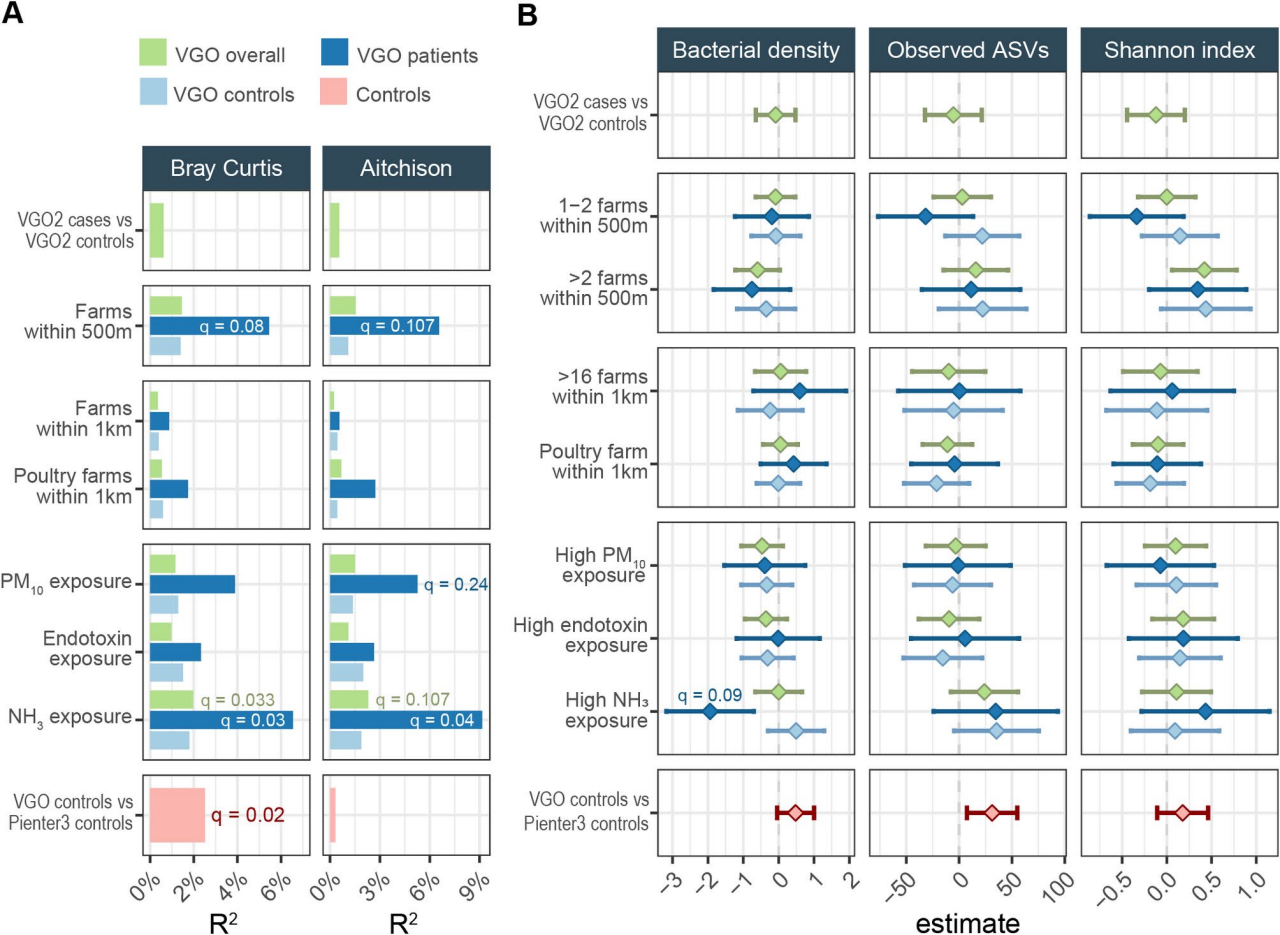
Beta and alpha diversity of the nasopharyngeal microbiota of COPD cases and controls living in proximity to livestock farms. (**A**) Explained variance of COPD status, livestock exposure-related characteristics and population (individuals living in a livestock dense area – VGO controls vs. the broader national population – Pienter3 controls) on nasopharyngeal microbial community composition, determined by multivariable PERMANOVA analyses based on Bray–Curtis dissimilarities with Hellinger transformation and Aitchison dissimilarities. (**B**) Linear regression results showing the relationship of bacterial density, observed ASVs and Shannon index (outcome) with COPD status, livestock exposure-related characteristics and population. Models were adjusted for season, age, smoking status, gender, education, atopy and antibiotics use. Coloured by group (VGO overall, VGO cases, VGO controls and Pienter3 controls). *q*-values were calculated using the Benjamini-Hochberg method to correct for multiple testing.

A multivariable PERMANOVA analysis, adjusted for season, age, smoking status, gender, education, atopy and antibiotics use, was used to compare the nasopharyngeal microbial community composition of individuals with COPD (VGO cases) to those without (VGO controls), showing no difference between groups (Fig. 2A; *R*^2^ = 0.6%; *q*-value > 0.25). Furthermore, when examining the impact of livestock farm exposure, we found that after adjusting for multiple testing, NH_3_ exposure (*q*-value = 0.03) and farms within 500 m (*q*-value = 0.08) remained important to variation in nasopharyngeal community composition in individuals with COPD. For the VGO controls, these effects were lower and not statistically significant. The Aitchison-based analysis supported the Bray-Curtis findings and additionally revealed a moderate association between PM₁₀ exposure and microbial community composition (Fig. 2A; *q*-value = 0.24).

Next, we examined bacterial density (measured by qPCR) and microbial diversity (determined by observed ASVs and the Shannon index) to further explore the influence of COPD status and the various farm exposure variables on the nasopharyngeal microbiota. Overall, the only association that remained after adjusting for multiple testing was a lower bacterial density in individuals with COPD exposed to high levels of NH_3_ (*q*-value = 0.09). Through a sensitivity analysis, we showed that NH_3_ was consistently associated with nasopharyngeal bacterial density in individuals with COPD, independent of the season of sampling. Interestingly, season of sampling was significantly associated with bacterial density in individuals without COPD but not in those with COPD (Fig. S4; *q*-value = 0.009). Although not statistically significant, this signal was accompanied by an increase in observed ASVs and Shannon index (Fig. 2B).

We also investigated whether individuals living in livestock-dense areas have a different nasopharyngeal microbiota compared to the broader national population and found a significant difference in community composition between VGO (representing individuals in livestock-dense areas) and Pienter3 controls (representing the broader Dutch population) (Fig. 2A; *R*_2_ = 2.9%; *q*-value = 0.02). Interestingly, both bacterial density and microbial diversity were higher in VGO compared to Pienter3 controls, though this difference was not statistically significant after adjusting for multiple testing (Fig. 2B; *q*-value > 0.25).

### Differential abundant taxa in the nasopharyngeal microbiota of individuals exposed to livestock farms with and without COPD.

We implemented two differential abundance analysis methods, MaAsLin2 and ANCOM-BC2, to examine the relationship of COPD status, NH_3_ exposure and number of farms within 500 m, as these factors were previously linked with changes in microbial community composition. We investigated the top 29 nasopharyngeal ASVs (> 1% relative abundance in at least 5% of the samples), together covering 82% of the relative abundance in all groups.

**Fig. 3:**
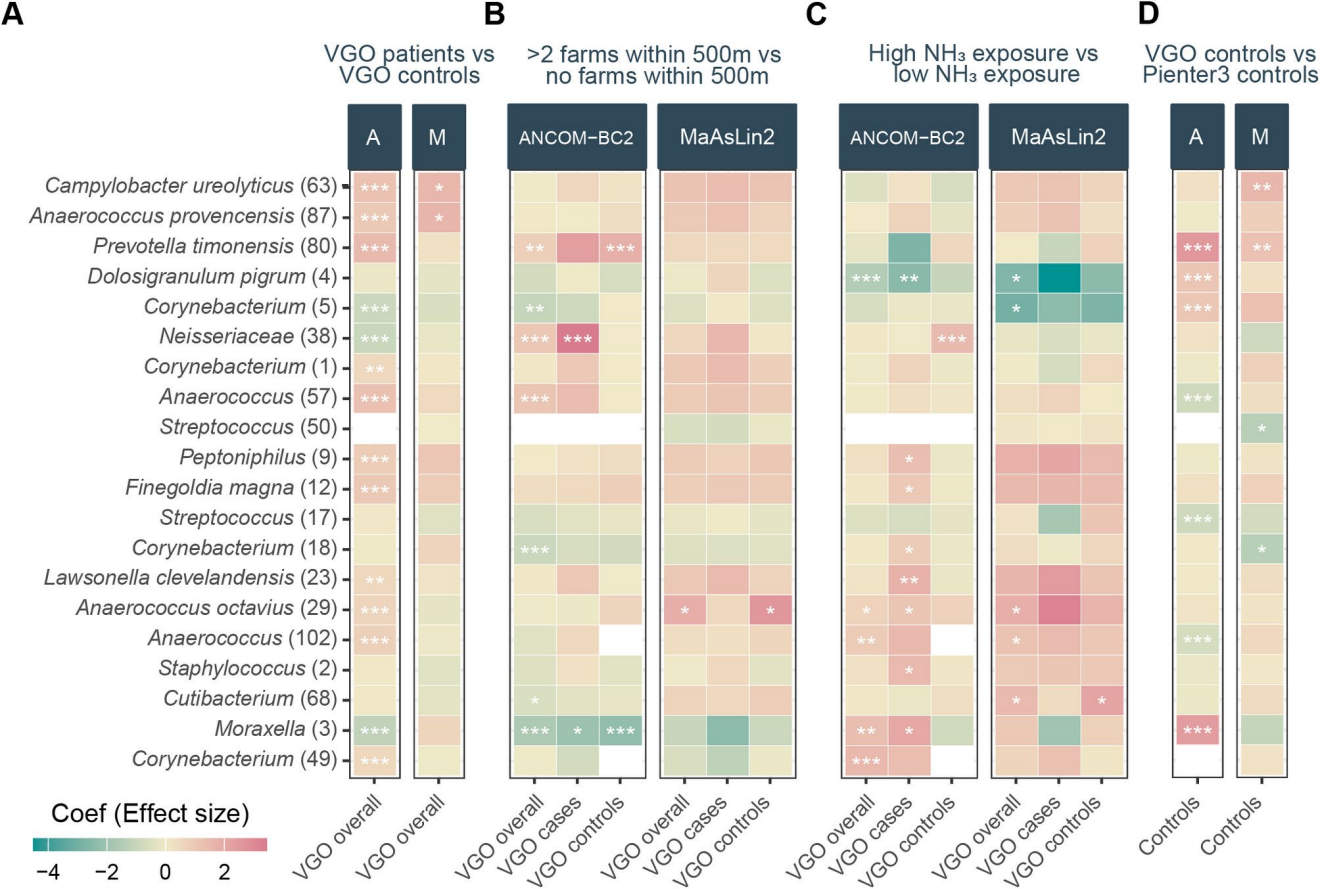
Differential abundance of the most abundant ASVs associated with COPD and livestock farming. Association between the absolute abundance of the 29 most abundant ASVs (> 1% relative abundance in at least 5% of the samples) and (**A**) COPD status (VGO cases vs. controls), (**B**) more than 2 farms within 500 m of residence, (**C**) high NH_3_ exposure, and (**D**) population (VGO controls vs. Pienter3 controls) as determined by ANCOM-BC2 - A and MaAsLin2 - M. The plot shows all the ASVs with at least one significant comparison. The colour corresponds to the direction of the association (pink for positive, blue for negative). Models were adjusted for season, age, smoking, gender, education, atopy and antibiotics use. q ≤ 0.25; *, q ≤ 0.1; **, q ≤ 0.05; ***. *q*-values were calculated using the Benjamini-Hochberg method to correct for multiple testing.

While COPD status did not significantly affect beta or alpha diversity, we observed subtle differences in the absolute abundance of the most prevalent ASVs. Notably, both ANCOM-BC2 and MaAsLin2 differential abundance analyses indicated higher absolute abundances of *Campylobacter ureolyticus* (63) and *Anaerococcus provensis* (87) in individuals with COPD compared to those without. Furthermore, nine, mostly rare anaerobic ASVs, including *Peptoniphilus* (9), *Prevotella timonensis* (80), *Anaerococcus* (57, 29 and 102), *Finegoldia magna* (12) and *Lawsonella clevelandensis* (23), had an increased absolute abundance in individuals with COPD as determined only by ANCOM-BC2. Importantly, typical nasopharyngeal ASVs *Moraxella* (3) and *Corynebacterium* (5), along with typical oral ASV *Neisseriaceae* (38) had a lower absolute abundance in individuals with COPD (Fig. 3A). A similar pattern was observed for *Moraxella* (3) and *Corynebacterium* (5) in individuals living within 500 m of more than two farms (Fig. 3B) and those with high NH_3_ exposure (Fig. 3C), respectively. Additionally, *Dolosigranulum pigrum* (4) exhibited lower absolute abundance in individuals exposed to high levels of NH_3_. When stratified by COPD status, this effect persisted only in those with COPD (Fig. 3C). The decrease in commensals was accompanied by a higher abundance of *Moraxella* (3) and an increase in rare ASVs, including *Cutibacterium* (68), *Anaerococcus octavius* (29), *Anaerococcus* (102) and *Corynebacterium* (49) in response to high levels of NH_3_ exposure in the overall VGO population. When stratified by COPD status, the majority of these effects were observed exclusively in individuals with COPD (Fig. 3C). Additionally, *Anaerococcus octavius* (29), *Anaerococcus* (57) and *Prevotella timonensis* (80) had a higher absolute abundance in individuals living close to more than two farms (Fig. 3B). Interestingly, ASVs with higher abundance in COPD and in response to farm exposure were generally classified as anaerobic bacteria.

When comparing VGO (rural) controls to Pienter3 (national) controls, the differential abundance analyses showed that the typical nasopharyngeal ASVs *Moraxella* (3), *D. pigrum* (4) and *Corynebacterium* (5) had a higher absolute abundance in VGO controls, whereas ASVs *Streptococcus* (17 and 50) and *Anaerococcus* (57 and 102) had a lower absolute abundance in VGO controls (Fig. 3D).

## Discussion

We aimed to explore the relationship between livestock-related air pollution – focusing on exposure to livestock-farming emitted PM_10_, endotoxin and NH_3_ and residential proximity to livestock farms - and the nasopharyngeal microbiota of individuals with COPD and healthy controls. Additionally, we characterized the stability of the nasopharyngeal microbiota over time and compared the nasopharyngeal microbial communities of individuals living in livestock-dense areas to those in the broader national population.

Interestingly, we found a higher absolute abundance of anaerobic bacteria, including *Peptoniphilus*, *Anaerococcus*,* Finegoldia magma* and *Prevotella*, alongside a lower abundance of common bacteria *Corynebacterium* and *Moraxella* in the nasopharynx of individuals with COPD when compared to those without. The prevalence of anaerobic bacteria in individuals with COPD, as observed in this study, aligns with findings from chronic rhinosinusitis (CRS) research, where higher abundances of anaerobes such as *Peptoniphilus*, *Anaerococcus*, and *Prevotella* had been reported^38–40^. This pattern may be attributed to several factors that foster anaerobic growth in chronic respiratory conditions. First, the selective pressure exerted by prolonged or recurrent antibiotic use in individuals with COPD could create an environment where anaerobes, which are often resistant to commonly used antimicrobial agents, can proliferate^41,42^. Second, the structural and functional changes in the airways of those with COPD, such as mucus hypersecretion and impaired mucociliary clearance, may create microenvironments that are low in oxygen^43^, providing hypoxic conditions favourable to the survival and expansion of anaerobic bacteria. Despite these findings, the interaction between anaerobes and the host immune response remains poorly understood, leaving it uncertain whether these bacteria directly contribute to disease progression or merely exploit the altered airway environment as opportunistic colonizers. Research on the nasopharyngeal microbiota in COPD remains scarce. Previous studies have identified alterations in the nasopharyngeal microbial communities^44,45^, with one study showing that particularly medication use (e.g., types of inhalers) was significantly associated with the microbial structure in nasal fluid of individuals with COPD^44^. A recent study showed that the airway of individuals with COPD provides a favourable environment for antimicrobial resistance genes, regardless of recent antibiotic use^46^, suggesting that frequent antibiotic use in COPD patients plays a significant role in the heightened prevalence of phenotypic antibiotic resistance^47^. Our study adds to the body of evidence by showing that specific members of the nasopharyngeal microbial community, particularly anaerobic bacteria, differ in abundance in individuals with COPD, suggesting a shift in these taxa as part of COPD-related microbial alterations.

Next, we focused on the potential differential effect of exposure to livestock farming in individuals with COPD compared to controls. Among the livestock-related exposure parameters studied, NH_3_ seemed to have the greatest influence on the nasopharyngeal microbial community composition. NH_3_ appeared as the primary driver, explaining 5.9% of the variation in the nasopharyngeal microbiota of individuals with COPD. Interestingly, we found that NH_3_ drives bacterial density changes in individuals with COPD, while seasonal factors play a more prominent role in individuals without COPD. The difference in microbiota structure was accompanied by a lower absolute abundance of *Dolosigranulum pigrum* and *Corynebacterium spp*. These commensal bacteria are deemed crucial, as their abundance is often linked to the absence of disease^48^ and inhibition of potential pathogens^49^. The observed decline in these beneficial microbes among those more exposed to livestock farming suggests a disruption in the protective microbial environment in individuals with COPD. Additionally, in the overall population, high exposure to NH_3_ was linked with an increase in anaerobic bacteria *Anaerococcus* and *Peptoniphilus*, which were previously linked with COPD. Interestingly, a study conducted in the Netherlands found that overall antibiotic use was higher in areas where residents lived in close proximity to poultry farms^50^. This observation suggests a potential correlation between residential exposure to livestock farms and increased antibiotic usage, which can lead to shifts in microbial communities and contribute to antibiotic resistance. Agriculture is responsible for over 81% of the global NH_3_ emissions^51^, highlighting its significant environmental impact. NH_3_ in the atmosphere acts as a precursor in the formation of secondary fine particulate matter (PM_2.5_)^52^, which is more directly linked to human health effects. PM_2.5_ can penetrate deeply into the lungs, leading to severe long-term health problems, including COPD and lung cancer^53,54^. Additionally, agricultural NH_3_ emissions have been associated with asthma development and progression in children^55,56^, underscoring the broader respiratory health risks connected to these emissions.

Our findings also revealed that living within 500 m of livestock farms was associated with a lower absolute abundance of *Moraxella* and a higher absolute abundance of *Anaerococcus* and *Prevotella*. To explore this further, we compared residents from livestock-dense areas (VGO controls) with those from the broader population (Pienter3 controls). We discovered that individuals in livestock-dense areas have a more diverse microbial community, as evidenced by a higher absolute abundance of typical nasopharyngeal microbes. This suggests that the environment in these regions may promote conditions that support a broader range of microbial species, potentially due to a higher variety of microorganisms in the air as a result of livestock emissions. Interestingly, a recent study suggested that the spatial distribution of livestock-related microbial agents can extend several kilometers from farms, implying that residents in these regions are likely exposed to a diverse array of microbial species^57^, further supporting our findings. Previous studies also reported that livestock workers had a higher nasal microbial diversity compared to non-livestock workers^58,59^. Our findings extend this observation, showing that the changes in nasal microbiota are not restricted to livestock workers but also affect individuals residing in close proximity to farms.

We observed greater concordance between consecutive nasopharyngeal samples from the same individuals compared to between individuals, suggesting that, like the oropharynx^27^, the nasopharynx serves as a suitable niche for studying environmental conditions and their impacts on microbial communities. Previous research has indicated that the nasopharynx may be more susceptible to environmental factors, such as seasonal changes, compared to other respiratory niches^25,60^, and can serve as an indicator of environmental exposure. Certain bacteria may not establish permanent colonization but can be transiently present due to airborne exposure, reflecting recent environmental conditions and microbial exposures. This underscores the significant, yet still not fully understood, role of the nasopharynx in disease progression, particularly under environmental influences like livestock farming.

A key strength of our study is the use of both a regional rural control group and a broader national population control, allowing us to account for local environmental factors while also providing a broader comparison group, enhancing the robustness of our findings on the effects of residential exposure to livestock farming. Nevertheless, it is important to note that the VGO and Pienter3 cohorts differ in both sampling protocols and collection periods, potentially introducing confounding from temporal changes in environmental conditions. Furthermore, variations in sampling, handling, and storage protocols between the cohorts may influence microbiome profiles, as microbial communities are highly sensitive to processing conditions. We tried to minimize this bias by processing all samples in the same laboratory using a controlled laboratory setting, where standardized operating procedures were rigorously applied to characterize low-biomass respiratory samples. This approach was crucial in ensuring the reliability of our data, as laboratory methods were specifically benchmarked to accommodate the unique challenges of low-biomass nasopharyngeal samples^29,48,61,62^. To further enhance data quality, we implemented extensive pre-processing steps to minimize contamination and exclude low-quality samples. Another limitation of the study is that it involved predominantly mild, population-based individuals with COPD. This group may exhibit less alterations in nasopharyngeal microbiota compared to individuals with more severe forms of COPD. In clinical settings, where they often experience more advanced stages of the disease, more significant shifts in the microbiota might be expected. For this reason, future studies might benefit from including a broader range of COPD severity, particularly focusing on more severe cases, to capture the full spectrum of nasopharyngeal microbiota variation associated with the disease.

By extending our previous work on the oropharyngeal microbiota^27^, we expand our understanding of the influence of environmental factors on microbial communities in different regions of the respiratory tract of individuals with COPD and those without. Our findings suggest that the altered nasopharyngeal environment in COPD cases, possibly influenced by antibiotic use and airway remodelling, may create conditions favourable to anaerobes. Furthermore, our research highlights the impact of residential exposure to livestock-related air pollutants, particularly NH_3_, on the nasopharyngeal microbiota. The significant association between NH_3_ exposure and shifts in microbial composition, especially the reduction of protective bacteria, underscores the broader implications of livestock farming emissions on respiratory health. Our research highlights the need to further unravel the complex interplay between livestock farming, chronic lung diseases and the role of the respiratory microbiota.

## Electronic supplementary material

Below is the link to the electronic supplementary material.

Supplementary Material 1

Supplementary Material 2

## Data Availability

Raw sequence data have been deposited in the Sequence Read Archive (SRA) at NCBI under accession number PRJNA1188511 (VGO) and PRJNA997934 (Pienter3). Scripts used in this study can be found on https://gitlab.com/Mari-Lee/NP_microbiota_COPD_Livestock.
