# Supplementary material for: Nasopharyngeal microbiota is influenced by agricultural air pollution in individuals with and without COPD
Source: Sci Rep. 2025 May 5;15:15653. doi: 10.1038/s41598-025-00242-9 (PMC12053623; doi:10.1038/s41598-025-00242-9)
Supplement: Supplementary file 1 — Supplementary Material 1 [file 41598_2025_242_MOESM1_ESM.pdf]

SUPPLEMENTARY DATA

Nasopharyngeal microbiota is influenced by agricultural air pollution in individuals with and without COPD

Mari-Lee Odendaal[1,2*], Julia Taenzer[1,2], Myrna M.T. de Rooij[2], Sjoerd Kuiling[1], Debby Bogaert[1,3,4], Eelco Franz[1], Lidwien A.M. Smit[2]

[1] Centre for Infectious Disease Control, National Institute for Public Health and the Environment (RIVM), Bilthoven, The Netherlands.
[2] Institute for Risk Assessment Sciences (IRAS), Utrecht University, Utrecht, the Netherlands.
[3] Department of Paediatric Immunology and Infectious Diseases, Wilhelmina Children's Hospital/University Medical Centre Utrecht, Utrecht, The Netherlands.
[4] Centre for Inflammation Research, Queen's Medical Research Institute, University of Edinburgh, Edinburgh, United Kingdom.

[*] Correspondence: mari.lee.odendaal@rivm.nl (M-L.O.)

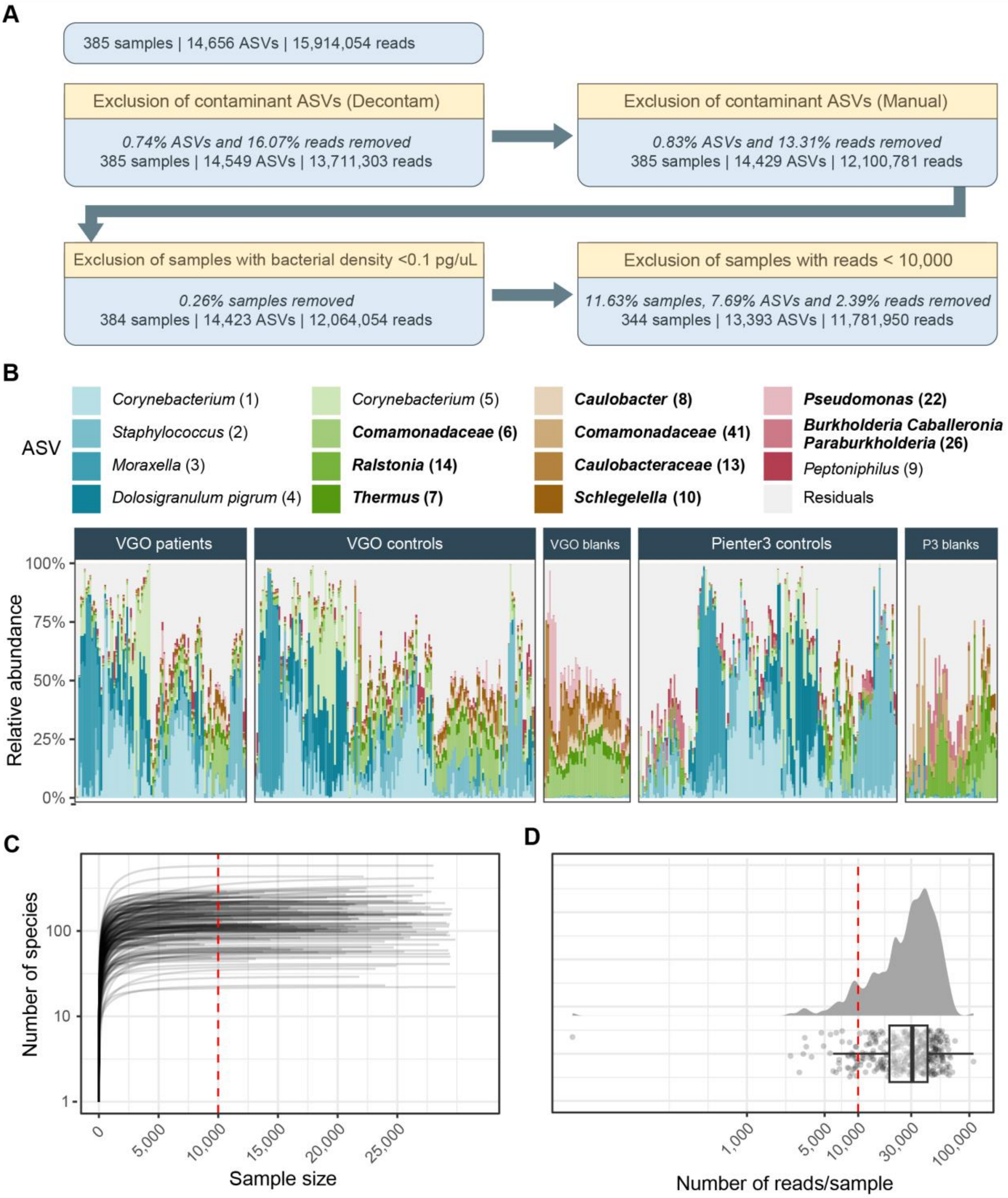

Fig. S1: Data pre-processing steps. A) Number of samples, ASVs and reads that were removed at each pre-processing step. B) The raw microbiota composition for the actual samples and isolation blanks from the VGO and Pienter3 population. Bold indicates ASVs identified as contaminants by the Decontam algorithm and manual filtering. C) Rarefaction curves for all the samples, showing that the microbial diversity has been adequately captured through the sequencing process. D) Raincloud plot of the distribution of the number of reads per sample. Samples with a read count of ≥10,000 reads were retained in our dataset.

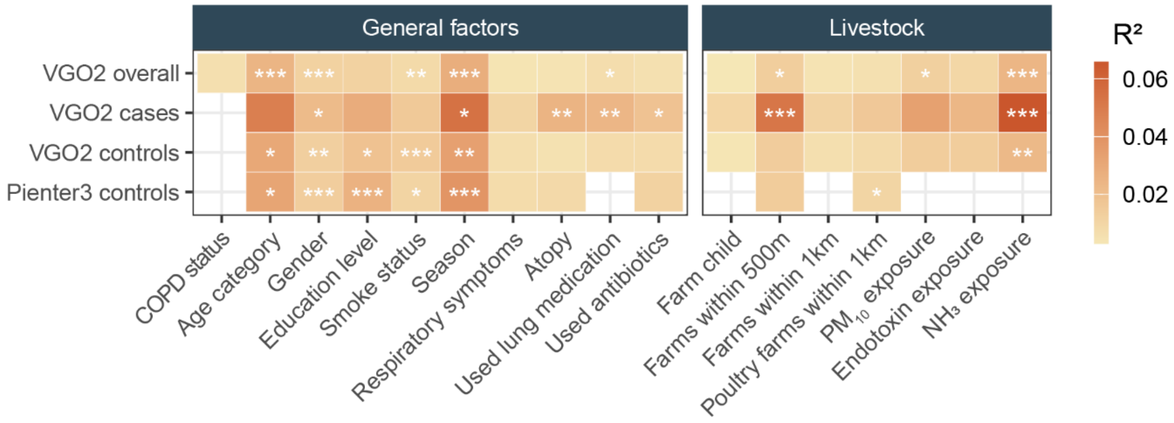

Fig. S2: Explained variance of general and livestock exposure-related characteristics on nasopharyngeal microbial community composition, determined by univariable PERMANOVA analyses based on Bray–Curtis dissimilarities with Hellinger transformation. p ≤ 0.25; *, p ≤ 0.1; **, p ≤ 0.05; ***. *Q*-values were calculated using the Benjamini-Hochberg (BH) method to correct for multiple testing.

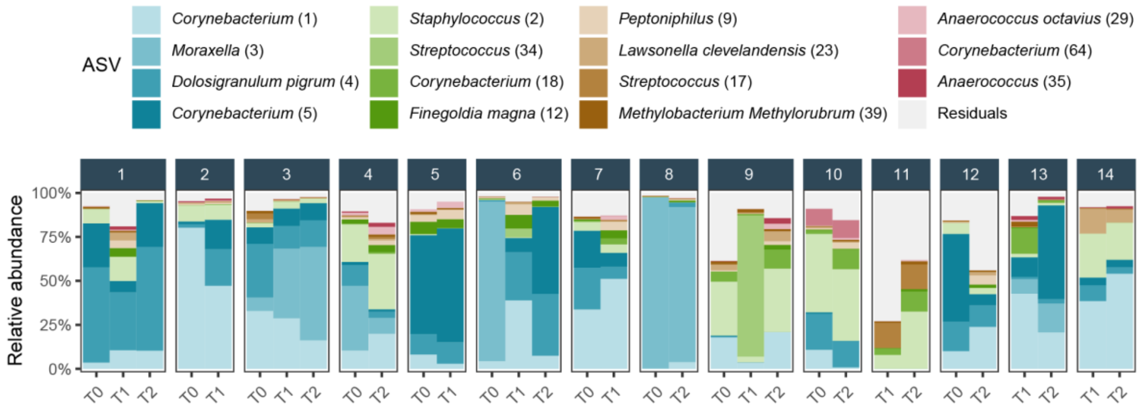

Fig. S3: Relative abundance of the top 15 ASVs across time points baseline (T0), 6 (T1) and 12 weeks (T2) for each individual with longitudinal data.

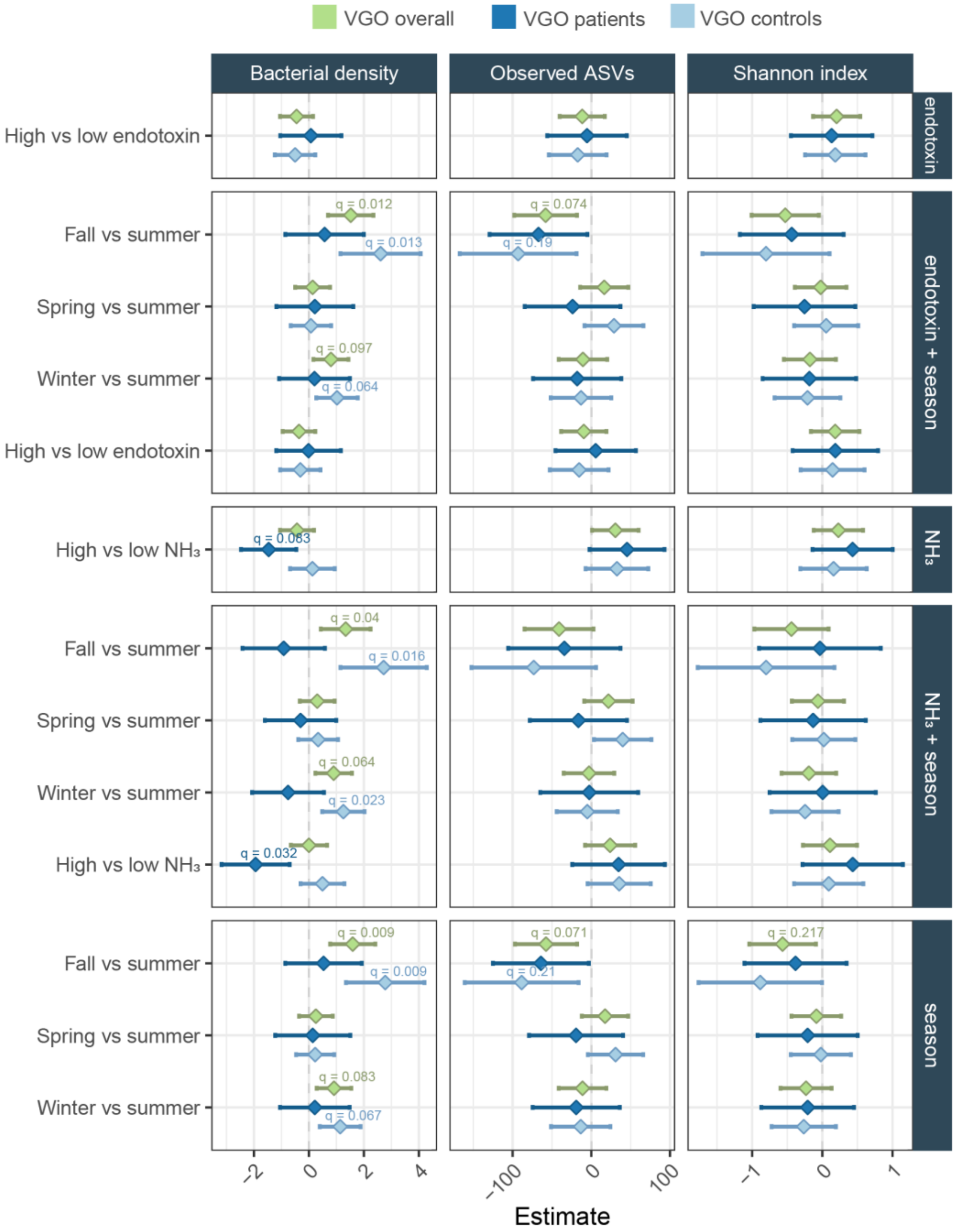

Fig. S4: Bacterial density and alpha diversity of the nasopharyngeal microbiota of COPD cases and controls living in proximity to livestock farms. Linear regression results showing the relationship of bacterial density, observed ASVs and Shannon index (outcome) with endotoxin, NH_3 and season. Models were adjusted for age, smoking status, gender, education, atopy and antibiotics use. Coloured by group (VGO overall, VGO cases and VGO controls). *Q*-values were calculated using the Benjamini-Hochberg (BH) method to correct for multiple testing.
